# Supplementary material for: Actinide Ion (Americium-241 and Uranium-232) Interaction with Hybrid Silica–Hyperbranched Poly(ethylene imine) Nanoparticles and Xerogels
Source: Gels. 2023 Aug 27;9(9):690. doi: 10.3390/gels9090690 (PMC10530514; doi:10.3390/gels9090690)
Supplement: Supplementary file 1 [file gels-09-00690-s001.zip › supplementary-gels-2572882.pdf]

Supplementary Information

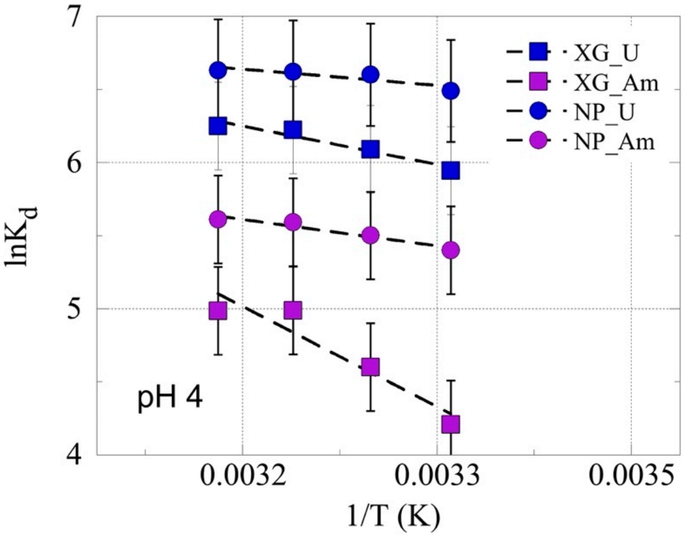

Figure S1. lnK$_d$ vs 1/T of the actinide ion adsorption by nanoparticles (NP) and xerogels (XG) at ultra-trace levels at pH 4. Experimental conditions: 10 mL of the solution, with 0.5 Bq/mL for both U-232 and Am-241 tracers, at different temperatures (25, 30, 35, 40 ^{0}C).

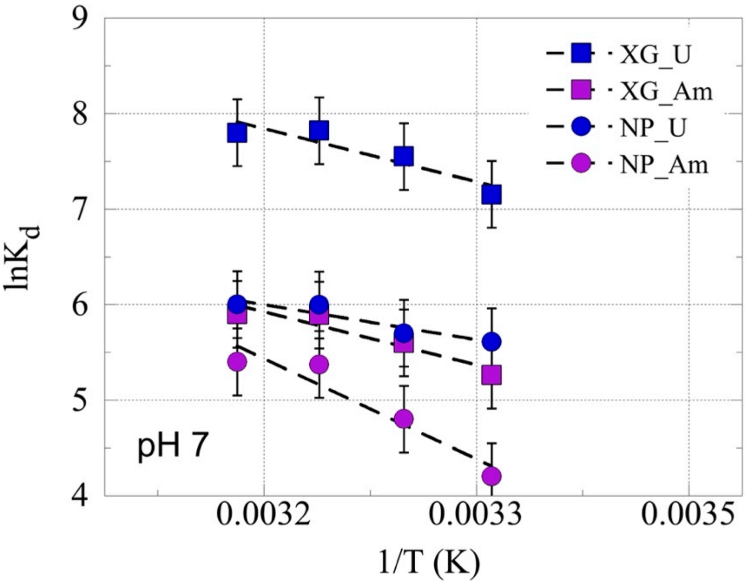

Figure S2. lnK$_d$ vs 1/T of the actinide ion adsorption by nanoparticles (NP) and xerogels (XG) at ultra-trace levels at pH 7. Experimental conditions: 10 mL of the solution, with 0.5 Bq/mL for both U-232 and Am-241 tracers, at different temperatures (25, 30, 35, 40 ^{0}C).

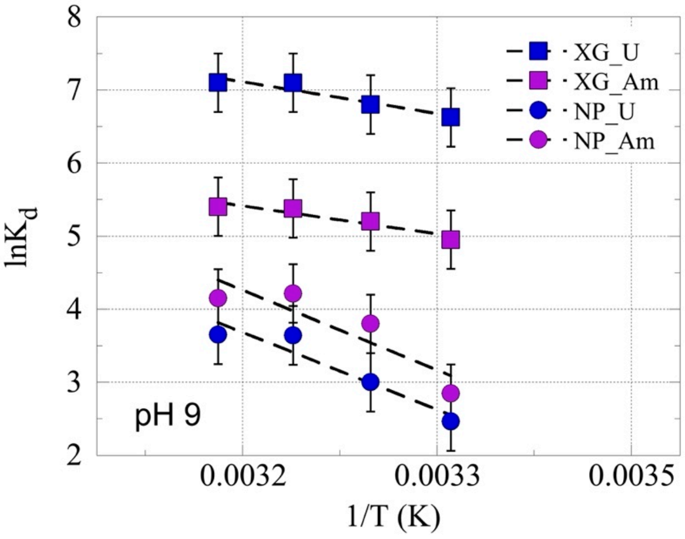

Figure S3. lnK_d vs 1/T of the actinide ion adsorption by nanoparticles (NP) and xerogels (XG) at ultra-trace levels at pH 9. Experimental conditions: 10 mL of the solution, with 0.5 Bq/mL for both U-232 and Am-241 tracers, at different temperatures (25, 30, 35, 40 ^{0}C).
